# Supplementary material for: Characterizing Glomerular Barrier Dysfunction with Patient-Derived Serum in Glomerulus-on-a-Chip Models: Unveiling New Insights into Glomerulonephritis
Source: Int J Mol Sci. 2024 May 8;25(10):5121. doi: 10.3390/ijms25105121 (PMC11121116; doi:10.3390/ijms25105121)
Supplement: Supplementary file 1 [file ijms-25-05121-s001.zip › ijms-2959982-supplementary.pdf]

Supplementary Table S1. General characteristics and treatment modalities of the IgAN.

	IgAN1	IgAN2	IgAN3	IgAN4	IgAN5
Age (yr)	39	60	36	64	46
Sex	female	female	male	female	male
SBP (mmHg)	130	151	130	120	121
DBP (mmHg)	85	100	50	64	71
WBC (10^3/uL)	8600	8600	9610	6740	8830
Hemoglobin (g/dL)	12.4	8.5	14.4	12	10.6
Platelet (10^3/uL)	244	131	247	301	181
UPCR (mg/g)	1827	4775.58	407.69	1084	637
Serum creatinine (mg/dL)	0.77	6.52	1.34	1.16	3.36
eGFR-MDRD (mL/min/1.73m^2)	88	6.6	62.6	48	20.1
Pathology					
S.M.K. LEE classification	II	V	IV	IV	IV
Oxford classification					
Mesangial hypercellularity (M)	1	0	1	1	1
Endocapillary hypercellularity (E)	0	0	0	0	0
Segmental glomerulosclerosis (S)	0	1	1	1	1
Tubular atrophy/interstitial fibrosis (T)	1	2	1	1	1
Cellular/ fibrocellular crescents (C)	0	0	0	0	0
IF					
Mesangial deposits of IgA	2+	2+	2+	3+	2+
Podocyte FPE	focal moderate	diffuse	focal mild	focal mild	focal mild

IgAN, Immunoglobulin A nephropathy; SBP, systoloic blood pressure; DBP, diastolic blood pressure; WBC, white blood cell; UPCR, urine protein/creatinine ratio; eGFR-MDRD, estimated glomerular filtration rate-modification of diet in renal disease; WHO, world health organization; IF, immunofluorescence; IgA, immunoglobulin A; FPE, foot process effacement.

Supplementary Table S2. General characteristics and treatment modalities of the MN.

	MN1	MN2	MN3	MN4	MN5
Age (yr)	40	62	55	82	80
Sex	female	male	female	female	male
SBP (mmHg)	134	135	136	111	138
DBP (mmHg)	77	86	89	68	80
WBC (10^3/uL)	5.8	5.42	8.8	5.4	8.62
Hemoglobin (g/dL)	12.6	15.9	13.6	13	10.9
Platelet (10^3/uL)	190	168	389	248	226
UPCR (mg/g)	10360.49	3312.62	9038.9	979.17	623.84
Serum creatinine (mg/dL)	0.67	0.7	0.71	1.02	0.99
eGFR-MDRD (mL/min/1.73m^2)	91	117.1	87.9	52.8	73.9
Anti-PLA2R antibody (RU/ml)	N/A	Negative	N/A	N/A	N/A
Pathology					
H&E staining					
Capillary wall thickening	diffuse	diffuse	diffuse	diffuse	diffuse
IF					
Glomerulus peripheral deposits IgG	3+	3+	3+	2+	3+
EM					
GBM thickening	diffuse	diffuse	diffuse	diffuse	diffuse
Podocyte FPE	diffuse	diffuse	diffuse	diffuse	diffuse
Subepithelial EDD	Large	Large	Moderate	Large	Large

MN, membranous nephropathy; SBP, systoloic blood pressure; DBP, diastolic blood pressure; WBC, white blood cell; UPCR, urine protein/creatinine ratio; eGFR-MDRD, estimated glomerular filtration rate-modification of diet in renal disease; RAS, renin-angiotensin system; MMF, mycophenolate mofetil; H&E, hematoxylin and eosin; IF, immunofluorescence; IgG, immunoglobulin G; EM, electron microscopy; GBM, glomerular basement membrane; FPE, foot process effacement; EDD, electron-dense deposits; N/A: not available.

Supplementary Table S3. General characteristics and treatment modalities of the MCD.

	MCD1	MCD2	MCD3	MCD4	MCD5
Age (yr)	77	52	40	52	53
Sex	female	female	male	male	female
SBP (mmHg)	134	122	145	155	138
DBP (mmHg)	64	68	82	90	85
WBC (10^3/uL)	6990	6110	9070	1740	8300
Hemoglobin (g/dL)	12.5	11.3	15	15	12.3
Platelet (10^3/uL)	357	227	276	310	188
UPCR (mg/g)	14618	1666	1199.88	10768.86	990.97
Serum creatinine (mg/dL)	0.66	0.47	0.95	0.93	0.78
eGFR-MDRD (mL/min/1.73m^2)	96.7	142.7	91.3	87.9	79.2
Pathology					
EM					
GBM thickening	normal	normal	normal	normal	normal
Podocyte FPE	diffuse	diffuse	focal marked	diffuse	focal marked

MCD, minimal change disease; SBP, systoloic blood pressure; DBP, diastolic blood pressure; WBC, white blood cell; UPCR, urine protein/creatinine ratio; eGFR-MDRD, estimated glomerular filtration rate-modification of diet in renal disease; RAS, renin-angiotensin system; EM, electron microscopy; GBM, glomerular basement membrane; FPE, foot process effacement.

Supplementary Table S4. General characteristics and treatment modalities of the LN.

	LN1	LN2	LN3	LN4	LN5
Age (yr)	32	79	42	30	77
Sex	Female	Female	Female	Female	Female
SBP (mmHg)	113	151	143	137	129
DBP (mmHg)	62	81	77	84	70
WBC (10^3/uL)	4800	6060	4300	3400	5860
Hemoglobin (g/dL)	12.1	10.8	10.9	8.4	10
Platelet (10^3/uL)	191	188	412	251	122
UPCR (mg/g)	3485.95	998.84	2295.75	7546.79	1937.78
Serum creatinine (mg/dL)	0.64	3.71	0.6	0.77	1.95
eGFR-MDRD (mL/min/1.73m^2)	115	12.1	114.4	94.6	25.4
FANA	+	+	+	+	+
SLE	-	+	+	-	+
Anti-dsDNA antibody (IU/mL)	11.8	11.4	343	34	5.1
C3 (mg/dL)	111	63.1	88.4	63.9	41.3
C4 (mg/dL)	28.5	38.4	18.9	24.8	10.4
Pathology					
S.M.K. LEE classification	V	N/A	IV	V	IV
IF	IgG/IgM/C3/C1q	No deposit	IgG/M/A/C3/C1q	IgG/M/A/C3/C1q	IgG/M/A/C3/C1q
EM					
GBM thickening	diffuse	normal	diffuse	diffuse	diffuse
Podocyte FPE	diffuse	focal mild	focal marked	diffuse	diffuse
Subepithelial EDD	Large	absent	Large	Large	Small

LN, lupus nephritis; SBP, systoloic blood pressure; DBP, diastolic blood pressure; WBC, white blood cell; UPCR, urine protein/creatinine ratio; eGFR-MDRD, estimated glomerular filtration rate-modification of diet in renal disease; FANA, fluorescent anti-nuclear antibody; SLE, systemic lupus erythematosus; Anti-dsDNA, anti-double stranded DNA; IgA, immunoglobulin A; IgG, immunoglobulin G; IgM, immunoglobulin M; C3, complement 3; C4, complement 4; C1q, complement 1q; IF, immunofluorescence; EM, electron microscopy; GBM, glomerular basement membrane; FPE, foot process effacement; EDD, electron-dense deposits; ; N/A: not available.
